# Microarray profile of human kidney from diabetes, renal cell carcinoma and renal cell carcinoma with diabetes

**DOI:** 10.18632/genesandcancer.51

**Published:** 2015-01

**Authors:** Adam Kosti, Hung-I Harry Chen, Sumathy Mohan, Sitai Liang, Yidong Chen, Samy L. Habib

**Affiliations:** ^1^ Geriatric Research, Education and Clinical Center, South Texas Veterans Healthcare System, University of Texas Health Science Center, San Antonio, Texas; ^2^ Department of Cellular & Structural Biology, University of Texas Health Science Center, San Antonio, Texas; ^3^ Department of Pathology, University of Texas Health Science Center, San Antonio, Texas; ^4^ Department of Greehey Children's Cancer Research Institute, University of Texas Health Science Center, San Antonio, Texas; ^5^ Department of Epidemiology and Biostatistics, University of Texas Health Science Center, San Antonio, Texas

**Keywords:** microarray, renal, diabetes, RCC

## Abstract

Recent study from our laboratory showed that patients with diabetes are at a higher risk of developing kidney cancer. In the current study, we have screened whole human DNA genome from healthy control, patients with diabetes or renal cell carcinoma (RCC) or RCC+diabetes. We found that 883 genes gain/163 genes loss of copy number in RCC+diabetes group, 669 genes gain/307 genes loss in RCC group and 458 genes gain/38 genes loss of copy number in diabetes group, after removing gain/loss genes obtained from healthy control group. Data analyzed for functional annotation enrichment pathways showed that control group had the highest number (280) of enriched pathways, 191 in diabetes+RCC group, 148 in RCC group, and 81 in diabetes group. The overlap GO pathways between RCC+diabetes and RCC groups showed that nine were enriched, between RCC+diabetes and diabetes groups was four and between diabetes and RCC groups was eight GO pathways. Overall, we observed majority of DNA alterations in patients from RCC+diabetes group. Interestingly, insulin receptor (INSR) is highly expressed and had gains in copy number in RCC+diabetes and diabetes groups. The changes in INSR copy number may use as a biomarker for predicting RCC development in diabetic patients.

## INTRODUCTION

Several epidemiological studies showed that diabetes is associated with an increased risk of cancer [1-8]. In addition, diabetes is also associated with a higher rate of mortality and cancer recurrence [8, 9]. A recent study from our laboratory showed that 25.4% of kidney cancer patients have diabetes (screening RCC patients from 1994-2009) indicting that diabetes is a major contributing factor in increasing the risk of kidney cancer [2]. Renal cell carcinoma (RCC) is a heterogeneous disease and is the most common malignancy of the adult kidney [1]. According to American Cancer Society report of 2014, kidney cancer is among the 10 most common cancers in both men and women. RCC has been shown to be very resistant to radiation or systemic chemotherapy [10]. The retrospective International Renal Cell Cancer study showed that a 5 to 10 year history of diabetes increased the relative risk for renal cancer by 40%, both in men and women [11]. In addition, the incidence and mortality rate of kidney cancer were increased by 47% and 43%, respectively, after age-adjusted [12].

HIF1/2A was found to be responsible for regulation the DNA damage response gene, DNA-damage-inducible transcript 4 (REDD1) in clear cell RCC (ccRCC) [13]. In addition, the same study showed that REDD1 inhibits tumor proliferation by interacting with the mTORC1 [13]. Sequencing analysis revealed that mutations in TSC1, TSC2 and REDD1 might result in inactivation of REDD1 and activation of mTORC1 [14]. On the other hand, VHL deficiency is a common feature of ccRCC and it is caused not only by VHL inactivation, but also by transcription elongation factor B polypeptide 1 (TCEB1) mutations [15]. TCEB1 prevents TCEB1-VHL binding and leading to overexpression of HIF in patients with ccRCC [15].

Several pathways and components recurrently mutated in ccRCC were detected by microarray analysis using human kidney tumor with ccRCC [16]. These mutations included PI3K-AKT-mTOR signaling and KEAP1-NRF2-CUL3 apparatus [14]. Recent study showed that gain of loci on 5q might provide evidence to be distinct role of VHL-HIF pathway in RCC carcinogenesis [17]. In addition, microarray profiling of human RCC samples revealed many differentially expressed genes in RCC (DEG) interacted with each other such as JAK2, IL8, BMPR2, FN1 and NCR1. These DEGs were significantly enriched in cytokine and cytokine receptors [15].

Microarray analysis of the RCC transcriptome revealed that many of up regulated genes were related to immune response and many of the down-regulated genes were related to oxidation-reduction [16]. In addition, significant difference in the immune responses gene expression in cytokine-cytokine pathways was identified between the normal kidney and RCC tissue [16]. Few chromosomal abnormalities have been also documented in RCC including VHL mutation (3p−), 5q21+ (70%), and 14q− (41%) [17, 18].

RCC is one of the most lethal cancers and approximately 50% of patients with metastatic RCC have a survival rate of less than one year [19]. Increased number of diabetic patients will increase the risk of solid tumors including renal cell carcinoma within those patients. Therefore, understanding the alterations at the genomic levels is essential to find a new biomarker for screening the diabetic subjects for early detection of cancer. To better understand the importance of genomic variations, DNA microarray was utilized in human kidney biopsies to find the gain/loss of genes and to determine the overlap in the enriched pathway(s) between diabetes, RCC and diabetes+RCC groups.

## RESULTS

### A. Screening genomic DNA from diabetes, RCC and RCC+diabetes patients for genes gain and loss

Screening of whole human genomic DNA in kidney tissues from 4 groups showed that 883 genes gain of copy number and 163 genes loss of copy number in RCC+diabetes (group 2), 669 genes gain and 307 genes loss in RCC (group 3), 458 gene gain and 38 loss in diabetes (group 4) after removing gain/loss genes obtained from healthy control group (group 1) (Table 1). Data of genome-wide screening showed that the highest number of genes with gain and loss in RCC+diabetes group compared to RCC and diabetes groups. In addition, RCC group showed higher gain and loss compared to diabetes group (Table 1).

**Table 1:** CNVs Observed in diabetes, RCC and RCC+diabetes versus control Group Screening the genome of kidney tissue revealed certain genes with CNVs. Those genes were then compared to control and any genes that overlapped were removed.

Group	Gain of Copy Number	Loss of Copy number
RCC & Diabetes (group 2)	883	163
RCC (group 3)	669	307
Diabetes (group 4)	458	38

**Figure 1:**
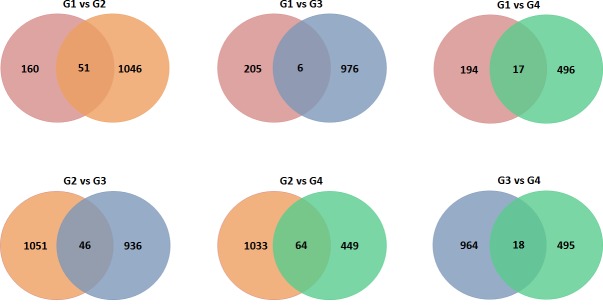
Copy Number Variations shared between all groups Screening the genome of kidney tissue revealed certain genes with CNVs. Venn Diagrams display the total number of shared genes between each group. Groups are as follows: Group 1-Control (Red), Group 2-RCC+diabetes (Orange), Group 3-RCC (Blue) and Group 4-diabetes (Green).

To future find the overlap between the 4 groups, we created Venn diagrams. Data in Figure 1 showed the sharing genes in pair-wise from all 4 groups. Data in Figure 1 indicated that higher CNVs overlap between RCC+diabetes and diabetes groups with 64 genes. These data also showed that there are 46 overlap genes between RCC+diabetes and RCC groups. On the other hand, lowest CNVs overlap was identified between control and RCC groups with total of 6 genes. Overall, the majority of CNVs overlapping between diabetes and diabetes+RCC groups suggesting that diabetes contribute in gain or loss of certain genes that may play a role in development of RCC in diabetic patient.

### B. Functional annotation enrichment analysis identifies Enriched Pathways

To better understand the differences of CNVs between the four groups, we used DAVID to identify enriched functional GO terms and biological pathways. The control group had the highest number of enriched pathways with total of 280 and only 28 pathways fell below a p-value of <0.05 (Table 2). In diabetes+RCC group, 191 pathways were enriched with more than half (107) fell below a p-value below <0.05. In diabetes group, 81 pathways were enriched and around half of them (41) having a p-value below <0.05. In RCC group, 148 pathways were enriched and less than half (75) of them have a p-value<0.05.

**Table 2:** DAVID identifies enriched functional terms and pathways in each group from genes with CNVs Total number of pathways was listed with DAVID setting a maximum p-value of 0.10.

Group	Functons Identified	Functions with (p<0.05)
Control (group 1)	280	28
RCC +Diabetes (group 2)	191	107
RCC (group 3)	148	75
Diabetes (group 4)	81	41

### C. Overlap functional terms and pathways between all groups

#### C.1. RCC+diabetes (Group 2) vs RCC (Group 3)

We found that nine pathways were enriched between RCC+diabetes and RCC groups (Table 3). The nine pathways identified were: positive regulation of protein modification process (GO:0031401), regulation of DNA replication (GO:0006275), positive regulation of macromolecule metabolic process (GO:0010604), positive regulation of nucleobase, nucleoside, nucleotide and nucleic acid metabolic process (GO:0045935), positive regulation of nitrogen compound metabolic process (GO:0051173), positive regulation of gene expression (GO:0010628), mitochondrial matrix (GO:0005759), mitochondrial lumen (GO:0031980), and organelle lumen (GO:0043233).

**Table 3:** Total number of genes with CNVs in each shared GO functions was compared between RCC+diabetes and RCC groups In addition p-values from DAVID were listed for each pathway between groups. Functional terms and pathways with p-value >0.05 were still included to avoid excluding biologically relevant information.

GO IDs	Gene Ontology	Number of Genes		p-value	
		RCC+Diabetes	RCC	RCC+Diabetes	RCC
GO:0031401	Positive regulation of protein modification process	18	12	0.005454	0.091633
GO:0006275	Regulation of DNA replication	9	6	0.007015	0.081179
GO:0010604	Positive regulation of macromolecule metabolic process	54	45	0.015281	0.019513
GO:0045935	Positive regulation of nucleobase, nucleoside, nucleotide and nucleic acid metabolic process	39	34	0.04338	0.027679
GO:0051173	Positive regulation of nitrogen compound metabolic process	40	35	0.046112	0.026344
GO:0010628	Positive regulation of gene expression	35	31	0.083903	0.044829
GO:0005759	Mitochondrial matrix	17	15	0.055564	0.059842
GO:0031980	Mitochondrial lumen	17	15	0.055564	0.059842
GO:0043233	Organelle lumen	97	83	0.064969	0.081846

#### C.2. RCC (Group 3) vs diabetes (Group 4)

DAVID revealed the following eight GO pathways are common between Diabetes and RCC alone: regulation of transcription (GO:0045449), transcription (GO:0006350), phosphate metabolic process (GO:0006796), phosphorus metabolic process (GO:0006793), nucleosome (GO:0000786), zinc ion binding (GO:0008270), chromosomal part (GO:0044427), and protein-DNA complex (GO:0032993) (Table 4).

**Table 4:** Genes with CNVs in each shared GO function and pathway was compared between RCC and diabetes In addition p-values from DAVID were listed for each pathway between groups. Pathways with p-value >0.05 were still included to avoid excluding biologically relevant information.

GO IDs	Gene Ontology	Number of Genes		P-value	
		Group 3	Group 4	Group 3	Group 4
GO:0045449	Regulation of transcription	121	75	0.024940517	0.017610183
GO:0006350	Transcription	97	65	0.031334602	0.004458755
GO:0006796	Phosphate metabolic process	47	31	0.040961577	0.051725116
GO:0006793	Phosphorus metabolic process	47	31	0.040961577	0.051725116
GO:0000786	Nucleosome	13	6	0.002917901	0.038220606
GO:0008270	Zinc ion binding	102	67	0.096811717	0.044543448
GO:0044427	Chromosomal part	26	16	0.076199416	0.025175795
GO:0032993	Protein-DNA complex	15	7	0.001804234	0.02992297

Six of the pathways involved in transcription regulation including, regulation of transcription, transcription, nucleosome, zinc ion binding, chromosomal part, and protein-DNA complex, are overlapped between RCC and diabetes (either regulating transcription or organizing the nucleus) (Table 4). The enrichment of CNVs of these six pathways suggests that it may have the same functions in diabetes and RCC.

#### C.3. RCC+diabetes (Group 2) vs diabetes (Group 4)

Overlap comparison between RCC+diabetes and diabetes groups revealed only four shared GO terms. These pathways are: response to cytokine stimulus (GO:0034097), ubiquitin-dependent protein catabolic process (GO:0006511), ATPase activity (GO:0016887), and adenyl nucleotide binding (GO:0030554) were found to be both enriched (Table 5). Recent gene expression microarray data of RCC samples revealed that genes involved in response to cytokine stimulus was overexpressed at the RNA level [15]. Our DAVID analysis revealed that the cytokine stimulus pathway was enriched at the DNA level in diabetic patients. For example Insulin receptor (INSR) had gain in RCC+diabetes and in diabetes groups. Interestingly, we found no change in copy number of INSR in the RCC group. The results from this DAVID analysis suggest that genomic changes in these functions like cytokine stimulus and INSR could contribute to renal tumor development in patients with diabetes. This suggests that diabetes may be involved in the development of RCC through genomic modifications of certain genes such as INSR.

**Table 5:** Genes with CNVs in each shared GO functions and pathways were compared between RCC+diabetes and diabetes In addition p-values from DAVID were listed for each function and/or pathway between groups. Pathways with p-value >0.05 were still included to avoid excluding biologically relevant information.

GO IDs	Gene Ontology	Number of Genes		p-value	
		Group 2	Group 4	Group 2	Group 4
GO:0034097	Response to cytokine stimulus	9	6	0.027668798	0.033227971
GO:0006511	Ubiquitin-dependent protein catabolic process	19	14	0.029376216	0.003496085
GO:0016887	ATPase activity	22	13	0.089531652	0.093114337
GO:0030554	adenyl nucleotide binding	84	46	0.098679016	0.09165851

### INSR is highly expressed in kidney sections of diabetic and RCC+diabetic patients

**Figure 2:**
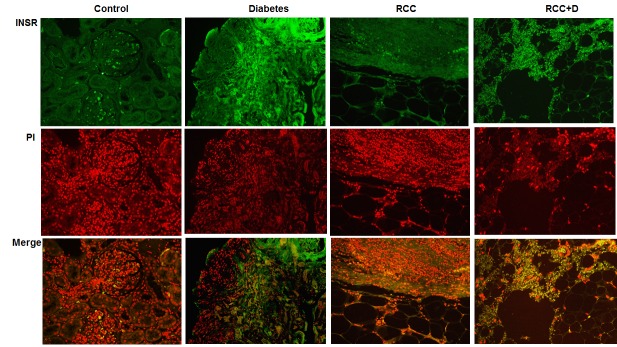
Immunostaining of INSR in kidney sections from control, diabetes, RCC, and RCC+diabetes FITC as green signals for INSR and propidium iodide (PI) red signals for nucleus showed the majority of staining of INSR in kidney sections of both diabetes and RCC+diabetes groups. Merge image showed the overlap of INSR with PI indicting strong staining of INSR in kidney sections from diabetes and RCC+D groups.

Immunofluorescence staining was performed in kidney sections from all four groups. Data in Figure 2 showed strong staining of INSR protein in kidney sections of RCC+diabetic and diabetes groups. On the other hand, normal healthy kidney section showed few cells staining of INSR within glomerular compartment. While very weak staining was detected in kidney section of RCC group (Fig. 2). These data suggest that increase copy number of INSR was associated with increase protein expression in diabetes and RCC+diabetes groups.

**Figure 3:**
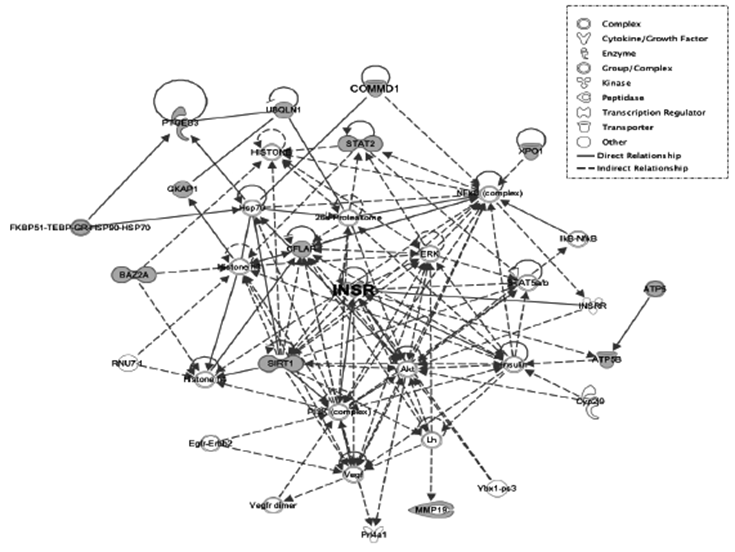
IPA network between RCC+diabetes and diabetes groups A network between RCC+diabetes and diabetes groups showed associated genes with INSR gene. Grey nodes indicate that those genes had observed CNVs and those genes are regulated by INSR. The node shapes denote complex, cytokine/growth factor, enzyme, kinase, peptidase, transcription regulator, transporter and other.

## DISCUSSION

Data from our study show that genome wide-analysis of kidney tissue samples from patients across four groups revealed many overlapping genes between patients with RCC+diabetes, diabetes and RCC. As diabetes increases the odds of developing RCC, it is likely that several pathways contribute to RCC development in diabetic patients. These data show that diabetes increases the genomic alterations in 496 genes with CNVs in comparison to healthy patients. On the other hand, the RCC group had almost double the number of copy number variations (976 CNVs). This is expected as CNVs can be dramatic events and changes in functional copy number could result in more frequent of CNVs in RCC group. A functional gain in BCL2, for example, could cause cells to become extremely resistant to apoptosis and resulting in proliferation and possibly oncogenesis. Importantly, we found increased CNVs in diabetic patients, which suggests that hyperglycemia may cause genomic alterations that could then result in gene dysregulation and potentially oncogenesis. In addition, adding diabetes to RCC (RCC+diabetes group) resulted in the most genomic variation with 1072 CNVs. This indicates that together RCC+diabetes had more effects on increase the genomic instability. We expect that the RCC+diabetes group will show the higher genomic variation since diabetes alone already caused increased copy number changes. To determine if there was any relationship between the CNVs observed in these groups, we used DAVID to identify GO function terms in common.

Chronic hyperglycemia, like in diabetes, can result in RCC development through diabetic nephropathy. We found that diabetes induces numerous genomic alterations in kidney tissue in comparison to healthy patients. DAVID analysis of the CNVs yielded shared pathways between diabetes and RCC groups. Of the eight pathways in common, six are involved in either transcriptional regulation (regulation of transcription (GO:0045449), transcription (GO:0006350), and zinc ion binding (GO:0008270) or nuclear organization (nucleosome (GO:0000786), chromosomal part (GO:0044427), protein-DNA complex (GO:0032993). The clustering of these pathways with similar functions suggested that both RCC and diabetes are related at the genomic level. We found that diabetes causes CNVs that are also found in RCC. However the impact of such copy number changes remain unclear, as not all genomic variations result in observable changes. Interestingly nucleosome pathway (GO:0000786) revealed that 13 histone-related genes had CNVs in RCC, while 6 CNVs were observed in diabetes patients. Histone dysregulation has been implicated in RCC development [21-23]. If diabetes does cause CNVs in these histone-nucleosome related genes, then it may contribute to RCC development and identifying candidate genes in these pathways may serve as a biomarker between diabetes and RCC.

Several genes in function terms and pathways overlap, including oxoglutarate dehydrogenase (OGDH), which forms a complex with its partner OGDHL to participate in AKT-signaling and NFKB function [24]. Our data show that OGDH in the broad GO function and pathways including mitochondrial lumen and organelle lumen which is important in degradation of glucose and glutamate. We found also gain in copy number of OGDH in RCC+diabetes and in RCC patients. On the other hand, we found that nine GO functions in common when comparing between RCC+diabetes and RCC using DAVID. Several of these function terms are broad such as mitochondrial matrix (GO:0005759) and mitochondrial lumen (GO:0031980). These data suggest that CNVs in mitochondria is common in RCC and RCC+diabetes. In addition, we found that several genes that interact with each other including PPP2R1A and PPP2CA, two phosphatases-associated genes involved in cell growth and division in regulation of DNA replication pathway (GO:0006275). It is known that DNA replication is an important step in cell division and lead the cells to proliferate at faster rate to initiate tumor. Therefore, CNVs in the regulation of DNA replication pathway may enable the cells to undergo rapid cell division and increase cell proliferation.

Four GO function terms were found to have direct overlap when comparing RCC+diabetes and diabetes group. In diabetes and RCC+diabetes groups, response to cytokine stimulus (GO:0034097) is enriched at the genomic level. These data indicates that diabetes causes genomic alterations that are observed in RCC at DNA level. Recent studies showed that response to cytokine stimulus (GO:0034097) is a pathway in RCC alone and cytokine pathways are enriched at the mRNA level [15, 16]. Another function term shared between both RCC+diabetes and diabetes is ubiquitin-dependent protein catabolic process (GO:0006511). Several genes associated with protein degradation are observed to have CNVs in both groups suggesting link between diabetes and RCC.

Interestingly, insulin receptor (INSR) a gene found in both responses to cytokine stimulus (GO:0034097) and ubiquitin-dependent protein catabolic process (GO:0006511) had gains in both RCC+diabetes and diabetes groups. Immunostaining data showed significant increase in INSR protein expression in kidney sections from diabetes and RCC+diabetes groups. These data suggested that increase in copy number of INSR is associated with increase expression of INRS protein in both groups. These changes in INSR copy number occurred more frequently in RCC+diabetes patients suggest that INSR can be consider a new biomarker to detect early renal tumor in diabetic patients. The Ingenuity Pathway Analysis (IPA) generated to show the Networks and Functional Analysis of INSR using shared copy number variations between diabetes and RCC+diabetes groups also suggest that INSR involved in direct or indirect regulation of several of kinases, enzymes, transporter and gene transcription that may play role in enhance tumorigenesis in diabetic patients. The regulation of these kinases by INSR is under investigation in our laboratory.

In summary, the overlaps between diabetes, RCC and RCC+diabetes groups in certain pathways suggest that these genomic changes may be contributed to develop RCC in diabetic patients. In addition, DNA microarray analysis revealed many CNVs with an increasing frequency in diabetes, RCC and RCC+diabetes groups. These data show for the first time that *INSR* copy number changes occur more frequently in RCC+diabetes group suggest that potential DNA alteration in *INSR* may play an important role in the early stages of tumor initiation. These data also suggest that INSR may a good candidate to be used as a biomarker for predicting RCC development in diabetic patients.

## MATERIALS AND METHODS

### Tissue Samples

Human kidney cortex tissues from de-identified cases of control (accidental death of healthy people), diabetes, and cancer with or without diabetes were obtained from the Tissue Bank for Development Disorders (University of Maryland, Baltimore, Maryland, USA) and San Antonio Cancer Institute Core, San Antonio, TX. Kidney biopsies from all groups were randomly selected from patients with age matched from 50-55 years old. Normal healthy subjects were identified with 5.0-5.5% of HgA1C. Diabetes was identified within the diabetic and diabetes+RCC groups with HgA1C ranged from 7.5-7.7%. Clear cell renal cell carcinoma was identified by Hematoxylin and Eosin staining in kidney sections from RCC and RCC+diabetes groups according to WHO criteria. The clinical database record did not contain complete information regarding the length of diabetes. The study has ethically approved by the Institutional Review Board of University of Texas Health Science Center at San Antonio, TX. DNA was extracted from the Formalin-fixed paraffin embedded (FFPE) samples (Life Technology, NY).

### Immunofluorescence staining of INSR

A fluorescent labeling method of INSR immunostaining was performed in kidney sections from all four groups as described previously (20). Fluorescein isothiocyanate (FITC) green signals for INSR were detected using a filter with excitation range 450–490 nm and propidium iodide (PI) red signals for nuclear DNA using a filter with excitation at 535 nm. Kidney sections were viewed and photographed using a Nikon Research microscope equipped for epifluorescence with excitation and band pass filters. To demonstrate staining specificity, control kidney sections were stained without primary antibody.

### aCGH Microarray Analysis

Agilent 8X60K CGH microarrays (Agilent Technologies, Inc., Santa Clara, CA) were performed to determine DNA copy number change of all patient samples following the manufacturer's protocol. Slides were scanned on an Agilent DNA Microarray Scanner, using standard settings. Microarray images were processed with Agilent Feature Extraction software to generate normalized, background-subtracted feature intensities. The microarray data were further analyzed with Nexus Copy number (BioDiscovery, Inc., Hawthorne, CA). In order to determine copy number variations (CNV), a log2 ratio threshold was set as (0.585/−0.8) for ‘gain/loss calls’. Aberrant segments from all samples with frequency higher than 25% were selected.

### Functional annotation enrichment analysis

To determine enriched pathways, genes with CNV were entered into Database for Annotation, Visualization and Integrated Discovery (DAVID) Functional Bioinformatics Tools (http://david.abcc.ncifcrf.gov/). Genes from autosomes were considered in the enrichment analysis. The default enrichment settings were used for testing enrichment of Gene Ontology (GO) terms from biological process, molecular function and cellular component, and pathways from KEGG and BIOCARTA. Pathways that were overlapping between groups were examined further to identify genes with an increase in copy number. DAVID evaluates the functional enrichment with a modified Fisher's exact test (EASE score). These p-values were used as guidelines for identifying pathways of interest, setting a maximum p-value of 0.10.

### Ingenuity Pathway Analysis (IPA)

Ingenuity Pathway Analysis (Ingenuity Systems, http://www.ingenutiy.com, Redwood City, CA) generated Canonical Pathways, Networks and Functional Analysis using shared copy number variations between diabetes and RCC+diabetes groups.

### Statistical analysis

DAVID calculated the significance of gene–term enrichment utilizing a modified Fisher's exact test (EASE score). These p-values were used as guidelines for identifying pathways of interest, setting a maximum p-value of 0.10.
